# Discovery of small protein complexes from PPI networks with size-specific supervised weighting

**DOI:** 10.1186/1752-0509-8-S5-S3

**Published:** 2014-12-12

**Authors:** Chern Han Yong, Osamu Maruyama, Limsoon Wong

**Affiliations:** 1 Graduate School for Integrative Sciences and Engineering, National University of Singapore, 28 Medical Drive, 117456 Singapore; 2 Institute of Mathematics for Industry, Kyushu University, Fukuoka, Japan; 3 School of Computing, National University of Singapore, 13 Computing Dr, 117417 Singapore

**Keywords:** protein complex, protein interaction, data integration, machine learning

## Abstract

The prediction of small complexes (consisting of two or three distinct proteins) is an important and challenging subtask in protein complex prediction from protein-protein interaction (PPI) networks. The prediction of small complexes is especially susceptible to noise (missing or spurious interactions) in the PPI network, while smaller groups of proteins are likelier to take on topological characteristics of real complexes by chance.

We propose a two-stage approach, SSS and Extract, for discovering small complexes. First, the PPI network is weighted by size-specific supervised weighting (SSS), which integrates heterogeneous data and their topological features with an overall topological isolatedness feature. SSS uses a naive-Bayes maximum-likelihood model to weight the edges with two posterior probabilities: that of being in a small complex, and of being in a large complex. The second stage, Extract, analyzes the SSS-weighted network to extract putative small complexes and scores them by cohesiveness-weighted density, which incorporates both small-co-complex and large-co-complex weights of edges within and surrounding the complexes.

We test our approach on the prediction of yeast and human small complexes, and demonstrate that our approach attains higher precision and recall than some popular complex prediction algorithms. Furthermore, our approach generates a greater number of novel predictions with higher quality in terms of functional coherence.

## Introduction

Most cellular processes are performed not by individual proteins acting alone, but by complexes consisting of multiple proteins that interact (bind) physically. Protein complexes comprise the modular machinery of the cell, performing a wide variety of molecular functions, so determining the set of existing complexes is important for understanding the mechanism, organization, and regulation of cellular processes. Since proteins in a complex interact physically, protein-protein interaction (PPI) data, made available in large amounts by high-throughput experimental techniques, is an important resource in the study of complexes. PPI data is frequently represented as a PPI network (PPIN), where vertices represent proteins and edges represent interactions between proteins. Protein complexes are groups of proteins that interact with one another, so they are usually dense subgraphs in PPI networks. Many algorithms have been developed to discover complexes from PPI networks based mainly on this idea [1-6].

It has been noted that the distribution of complex sizes follows a power law distribution [7], meaning that a large majority of complexes are small. Thus the discovery of small complexes is an important subtask within complex discovery. An inherent difficulty is that the strategy of searching for dense clusters becomes problematic: fully dense (ie. cliques) size-2 and size-3 clusters correspond to edges and triangles respectively, and only a few among the abundant edges and triangles of the PPI network represent actual small complexes. Furthermore, high-throughput PPI data suffers from significant amounts of noise, in terms of false positives (spuriously detected interactions) as well as false negatives (missing interactions). This presents a challenge for complex discovery from PPI data, and is especially severe for the discovery of small complexes, which is much more sensitive to extraneous or missing edges: for a size-2 complex, a missing co-complex interaction disconnects its two member proteins, while only two extraneous interactions are sufficient to embed it within a larger clique (a triangle).

Our proposed approach to address these challenges consists of two steps. First, we weight the edges of the PPI network with the probabilities of belonging to a complex, in a size-specific manner. Second, we extract the small complexes from this weighted network. In the first step, our weighting approach, called size-specific supervised weighting (SSS), integrates three different data sources (PPIs, functional associations, and literature co-occurrences) with their topological characteristics (degree, shared neighbours, and connectivity between neighbours), as well as an overall topological isolatedness feature. SSS uses a supervised maximum-likelihood naive-Bayes model to weight each edge with two separate probabilities: that of belonging to a small complex, and of belonging to a large complex. In the second step, our complex extraction approach, called Extract, uses these weights to predict and score candidate small complexes, by weighting their densities with a cohesiveness function [5] that incorporates both small and large co-complex probabilities of edges within and around each cluster.

In our previous approach, Supervised Weighting for Composite Networks (SWC [8]), we integrated diverse data sources (including topological characteristics) with a supervised approach to accurately score edges with co-complex probabilities, and attained good performance in predicting large complexes (of size greater than three) in yeast and human. However, SWC's performance in scoring edges from small complexes is unsatisfactory. This is because edges in small complexes have radically different topological characteristics from edges in large complexes. And since there are a far greater number of edges from large complexes than from small complexes, the learned model reflects the features of the former rather than the latter. Thus, here we use a model for small complexes specifically, which captures the characteristics of their edges more accurately.

By integrating two additional data sources (functional associations and literature co-occurrences) with supervised learning, our approach reduces the amount of spurious interactions among the PPIs. Complexes tend to be characterized by certain topological characteristics in the PPI network (for example, they tend to be densely connected and bordered by a sparse region), but smaller groups of proteins are likelier to take on such characteristics by chance. Integrating topological features from multiple data sources reduces the discovery of false positive complexes, as it is less likely that all data sources share such characteristics by chance in a random set of proteins.

An important topological characteristic of complexes is that they tend to be topologically isolated, or bordered by a sparse region. Many complexes exhibit a core-attachment structure [9], where distinct complexes can share common subsets of proteins (called the core), with variations among the remaining proteins (attachments). Since distinct complexes can share proteins, they overlap in the PPI network, and thus are not expected to be completely isolated; nonetheless, proteins in small complexes with core-attachment structures are still more isolated than those in large complexes. Thus we incorporate an isolatedness feature derived from an initial posterior probability calculation, which contributes to discriminating between edges in small complexes, large complexes, or in no complex.

Predicted complexes are typically given some score indicative of confidence in the prediction. The weighted density of the predicted complex is frequently used for this purpose (for example in [4,8]): assuming the edge weights represent co-complex estimates, the weighted density averages over the weights of all the edges within the predicted complex, giving an overall measurement of the prediction's reliability. However, for predicted small complexes the weighted density is derived from only one or three edges (corresponding to size-2 or size-3 clusters respectively), making it susceptible to noisy edge weights. Thus we incorporate a cohesiveness function in scoring predicted complexes, which includes both internal edges within the cluster, as well as outgoing edges around the cluster.

We test our approach on the prediction of small complexes in yeast and human, and obtain improved performance in both organisms. In the rest of the paper, we first describe each of the two steps of our approach. Next we describe our experimental methodology, and finally present and discuss our results.

## Methods

In this section, we describe our approach for predicting small protein complexes, which consists of two stages: first, size-specific supervised weighting (SSS) of the PPIs; second, extracting small complexes from this weighted PPI network.

### Size-specific supervised weighting (SSS) of the PPI network

SSS uses supervised learning to weight each edge of the reliable PPI network with two posterior probabilities, that of being a small-co-complex edge (ie. of belonging to a small complex), and that of being a large-co-complex edge, given the edge's features. These features consist of diverse data sources, their topological characteristics, and an isolatedness feature derived from an initial calculation of the posterior. We first describe the data sources and features we use, then describe our weighting approach.

#### Data sources and features

We use three different data sources (PPI, functional association, and literature co-occurrence) together with their topological characteristics as features. Each data source provides a list of scored protein pairs: for each pair of proteins (*a, b*) with score *s, a *is related to *b *with score *s*, according to that data source. For both yeast and human, the following data sources are used:

• *PPI *: PPI data is obtained by taking the union of physical interactions from BioGRID [10], IntAct [11] and MINT [12] (data from all three repositories downloaded in January 2014). In addition, in yeast we also incorporate the widely-used Consolidated PPI dataset [13]. We unite these datasets, and score and filter the PPIs, using a simple reliability metric based on the Noisy-Or model to combine experimental evidences (also used in [14]). For each experimental detection method *e*, we estimate its reliability as the fraction of interactions detected where both interacting proteins share at least one high-level cellular-component Gene Ontology term. Then the reliability of an interaction (*a, b*) is estimated as:

$$reliability\left(a,b\right)=1-\underset{i\in E_{a,b}}{\prod }{\left(1-rel_{i}\right)}^{n_{i,a,b}}$$

where *rel_i _*is the estimated reliability of experimental method *i, E_a,b _*is the set of experimental methods that detected interaction (*a, b*), and *n_i,a,b _*is the number of times that experimental method *i *detected interaction (*a, b*). The scores from the Consolidated dataset are discretized into ten equally-spaced bins (0*−*0.1, 0.1*−*0.2*, . . *.), each of which is considered as a separate experimental method in our scoring scheme. We avoid duplicate counting of evidences across the datasets by using their publication IDs (in particular, PPIs from the Consolidated dataset are removed from the BioGRID, IntAct, and MINT datasets).

• *STRING *: Predicted functional association data is obtained from the STRING database [15] (data downloaded in January 2014). STRING predicts each association between two proteins *a *and *b *(or their respective genes) using the following evidence types: gene co-occurrence across genomes; gene fusion events; gene proximity in the genome; homology; co-expression; physical interactions; co-occurrence in literature; and orthologs of the latter five evidence types transferred from other organisms (STRING also includes evidence obtained from databases, which we discard as this may include co-complex relationships which we are trying to predict). Each evidence type is associated with quantitative information (e.g. the number of gene fusion events), which STRING maps to a confidence score of functional association based on co-occurrence in KEGG pathways. The confidence scores of the different evidence types are then combined probabilistically to give a final functional association score for (*a, b*). Only pairs with score greater than 0.5 are kept.

• *LIT *: Co-occurrence of proteins or genes in PubMed literature (data down-loaded in June 2012). Each pair (*a, b*) is scored by the Jaccard similarity of the sets of papers that *a *and *b *appear in:

$$s=\frac{\left|A_{a}\cap A_{b}\right|}{\left|A_{a}\cup A_{b}\right|}$$

where *A_x _*is the set of PubMed papers that contain protein *x*. For yeast, that would be the papers that contain the gene name or open reading frame (ORF) ID of *x *as well as the word "cerevisiae"; for human that would be the papers that contain the gene name or Uniprot ID of *x *as well as the words "human" or "sapiens".

For each protein pair in each data source, we derive three topological features: degree (DEG), shared neighbors (SHARED), and neighborhood connectivity (NBC). For each data source, the edge weight used to calculate these topological features is the data source score of the edge.

• *DEG *: The degree of the protein pair (*a, b*), or the sum of the scores of the outgoing edges from the pair:

$$DEG\left(a,b\right)=\underset{x\in N_{a}\backslash \left\{b\right\}}{\sum }w\left(a,x\right)+\underset{x\in N_{b}\backslash \left\{a\right\}}{\sum }w\left(b,x\right)$$

where *w*(*x, y*) is the data source score of edge (*x, y*), *N_a_* is the set of all neighbours of *a*, excluding *a*.

• *NBC *: The neighborhood connectivity of the protein pair (*a, b*), defined as the weighted density of all neighbors of the protein pair excluding the pair themselves:

$$NBC\left(a,b\right)=\frac{\underset{x,y\in N_{a,b}}{\sum }w\left(x,y\right)}{\text{min}\left(|N_{a,b}|,\lambda \right)\left(\text{min}(|N_{a,b}|,\lambda \right)-1)}$$

where *w*(*x, y*) is the data source score of edge (*x, y*); *N_a,b _*is the set of all neighbours of *a *and *b*, excluding *a *and *b *themselves; *λ *is a dampening factor.

• *SHARED *: The extent of shared neighbors between the protein pair, derived using the Iterative AdjustCD function (with two iterations) [4].

This gives a total of twelve features: the three data sources *PPI, STRING*, and *LIT *, and nine topological features (three for each data source), *DEG_PPI _, DEG_STRING_, DEG_LIT _, SHARED_PPI _, SHARED_STRING_, SHARED_LIT _, NBC_PPI _, NBC_STRING_*, and *NBC_LIT _*. In addition, a feature called isolatedness is incorporated after an initial calculation of the posterior probabilities, as described below.

#### Size-specific supervised weighting of the PPI network (SSS)

In this step, we weight the edges of the PPI network with our size-specific supervised weighting (SSS) approach. We use a highly-reliable subset of the PPI network, by keeping only the top *k *edges with the highest PPI reliability scores. In our experiments we set *k *= 10000, but similar results are obtained for other values of *k*. SSS uses supervised learning to weight each edge with three scores: its posterior probability of being a small-co-complex edge (ie. of belonging to a small complex), of being a large-co-complex edge, and of not being a co-complex edge, given the features of the edge. These features consist of the twelve features described above (*PPI, STRING, LIT *, and nine topological features), as well as an isolatedness feature which is derived from an initial calculation of the posterior probabilities. We use a naive-Bayes maximum-likelihood model to derive the posterior probabilities.

**Figure 1:**
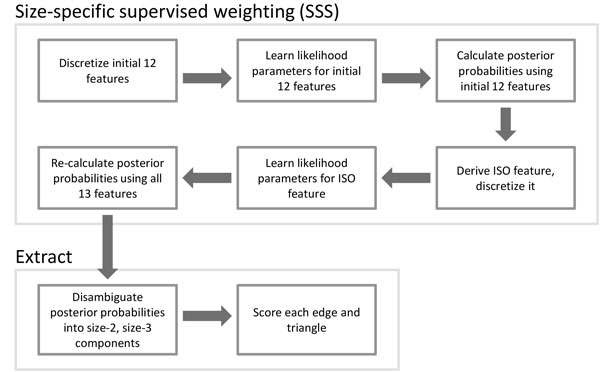
**Flowchart of our approach consisting of size-specific supervised weighting (SSS) and Extract**.

Each edge (*a, b*) in the network is cast as a data instance, with its set of features **F**. Using a reference set of protein complexes, each edge (*a, b*) in the training set is given a class label *lg-comp *if both *a *and *b *are in the same large complex; it is labelled *sm-comp *if both *a *and *b *are in the same small complex; otherwise it is labelled *non-comp*. Learning proceeds by the following steps (illustrated in Figure 1):

1 Minimum description length (MDL) supervised discretization [16] is performed to discretize the features (excluding the isolatedness feature). MDL discretization recursively partitions the range of each feature to minimize the information entropy of the classes. If a feature cannot be discretized, that means it is not possible to find a partition that reduces the information entropy, so the feature is removed. Thus this step also serves as simple feature selection.

2 The maximum-likelihood parameters are learned for the three classes *lg-comp, sm-comp*, and *non-comp*:

$$P\left(F=f|sm\text{-}comp\right)=\frac{n_{sm,F=f}}{n_{sm}}$$

$$P\left(F=f|lg\text{-}comp\right)=\frac{n_{lg,F=f}}{n_{lg}}$$

$$P\left(F=f|non\text{-}comp\right)=\frac{n_{non,F=f}}{n_{non}}$$

for each discretized value *f *of each feature *F *(excluding the isolatedness feature). *n_sm _*is the number of edges with class label *sm-comp, n_sm,F = f _*is the number of edges with class label *sm-comp *and whose feature *F *has value *f ; n_lg _*is the number of edges with class label *lg-comp, n_lg,F = f _*is the number of edges with class label *lg-comp *and whose feature *F *has value *f ; n_non _*is the number of edges with class label *non-comp*, and *n_non,F = f _*is the number of edges with class label *non-comp *and whose feature *F *has value *f *.

3 Using the learned models, the class posterior probabilities are calculated for each edge (*a, b*) using the naive-Bayes formulation:

$$P\left(\left(a,b\right)\;is\;sm\text{-}comp|F_{1}=f_{1},F_{2}=f_{2},...\right)=\frac{\underset{i}{\prod }P\left(F_{i}=f_{i}|\left(a,b\right)is\;sm\text{-}comp\right)P\left(sm\text{-}comp\right)}{\sum_{class\in \left\{sm\text{-}comp,lg\text{-}comp,non\text{-}comp\right\}}\underset{i}{\prod }P\left(F_{i}=f_{i}|\left(a,b\right)is\;class\right)P\left(class\right)}$$

The posterior probabilities are calculated in a similar fashion for the other two classes *lg-comp *and *non-comp*. We abbreviate the posterior probability of edge (*a, b*) being in each of the three classes as *P*_(*a,b*)*,sm*_, *P*_(*a,b*)*,lg *_, and *P*_(*a,b*)*,non*_.

4 A new feature ISO (isolatedness) is calculated for each edge (*a, b*), based on the probability that the edge is isolated (not adjacent to any other edges), or is part of an isolated triangle:

ISO(a,b)=ISO2(a,b)+ISO3(a,b)

$$\begin{array}{c} ISO2\left(a,b\right)=P_{\left(a,b\right),sm}\underset{x\in \left\{a,b\right\},y\in N_{a,b}}{\prod }P_{\left(x,y\right),non} \end{array}$$

$$ISO3\left(a,b\right)=\underset{c\in N_{a}\cap N_{b}}{\sum }\left(P_{\left(a,b\right),sm}P_{\left(a,c\right),sm}P_{\left(b,c\right),sm}\underset{x\in \left\{a,b,c\right\},y\in N_{a,b,c}}{\prod }P_{\left(x,y\right),non}\right)$$

where *N_x _*denotes the neighbours of *x*, excluding *x*. The ISO feature is discretized with MDL.

5 The maximum-likelihood parameters for the ISO feature are learned for the three classes.

6 The posterior probabilities for the three classes, *P*_(*a,b*)*,sm*_, *P*_(*a,b*)*,lg *_, and *P*_(*a,b*)*,non*_, are recalculated for each edge (*a, b*), this time incorporating the new ISO feature.

### Extracting small complexes

After using SSS to weight the PPI network, the small complexes are extracted. This stage, called Extract, consists of two steps (see Figure 1): first, the small-co-complex probability weight of each edge is disambiguated into size-2 and size-3 complex components; next, each candidate complex is scored by its cohesiveness-weighted density, which is based on both its internal and outgoing edges.

In the disambiguation step, the small-co-complex probability weight of each edge (*a, b*) = *P*_(*a,b*)*,sm*_, which denotes the probability of being in a small (either size-2 or size-3) complex, is decomposed into two component scores (we use the term score instead of probability since its derivation is not probabilistic): $P_{\left(a,b\right),sm2}^{\prime }$, which is the score of being in the size-2 complex composed of *a *and *b*; and $P_{\left(a,b\right),sm3,abc}^{\prime }$, which is the score of being in the size-3 complex composed of *a, b*, and *c*. Intuitively, if an edge is contained within a triangle with high edge weights, then it is likelier to be a size-3 complex corresponding to the triangle rather than a size-2 complex; thus its size-2 component score should be reduced based on the weights of incident triangles:

$${P^{\prime }}_{\left(a,b\right),sm2}=P_{\left(a,b\right),sm}-\sum_{x\in N_{a}\cap N_{b}}P_{\left(a,b\right),sm}P_{\left(a,x\right),sm}P_{\left(b,x\right),sm}$$

Similarly, if an edge is contained within a triangle with high edge weights, and is also within another triangle with low edge weights, then it is likelier to form a size-3 complex with the former triangle rather than the latter; thus its size-3 component score corresponding to a specific triangle should be reduced based on the weights of its other incident triangles:

$$P_{\left(a,b\right),sm3,abc}^{\prime }=P_{\left(a,b\right),sm}-\sum_{x\in N_{a}\cap N_{b}\backslash \left\{c\right\}}P_{\left(a,b\right),sm}P_{\left(a,x\right),sm}P_{\left(b,x\right),sm}$$

In the next step, each candidate complex is scored by weighting the density of the cluster with its cohesiveness, which is adapted from cluster cohesiveness as described in [5]. Here, we define cohesiveness of a cluster as the ratio of the sum of its internal edges' weights over its internal plus outgoing edges' weights, where the internal weights are the component scores as calculated above, and the external weights are the posterior probabilities of being either small or large co-complex edges. The cohesiveness of a size-2 cluster (*a, b*) and a size-3 cluster (*a, b, c*) respectively are:

$$Coh\left(a,b\right)=\frac{{P^{\prime }}_{\left(a,b\right),sm2}}{{P^{\prime }}_{\left(a,b\right),sm2}+\underset{x\in \left\{a,b\right\},y\in N_{a,b}}{\sum }\left(P_{\left(x,y\right),sm}+P_{\left(x,y\right),lg}\right)}$$

$$Coh\left(a,b,c\right)=\frac{{P^{\prime }}_{\left(a,b\right),sm3,abc}+{P^{\prime }}_{\left(a,c\right),sm3,abc}+{P^{\prime }}_{\left(b,c\right),sm3,abc}}{{P^{\prime }}_{\left(a,b\right),sm3,abc}+{P^{\prime }}_{\left(a,c\right),sm3,abc}+{P^{\prime }}_{\left(b,c\right),sm3,abc}+\underset{x\in \left\{a,b,c\right\},y\in N_{a,b,c}}{\sum }\left(P_{\left(x,y\right),sm}+P_{\left(x,y\right),lg}\right)}$$

We then define the score of a cluster as its cohesiveness-weighted density, or the product of its weighted density and its cohesiveness. The score of a size-2 cluster (*a, b*), and a size-3 cluster (*a, b, c*) respectively are:

$$score\left(a,b\right)=Coh\left(a,b\right)P_{\left(a,b\right),sm2}^{\prime }$$

$$score\left(a,b,c\right)=Coh\left(a,b,c\right)\frac{\left({P^{\prime }}_{\left(a,b\right),sm3,abc}+{P^{\prime }}_{\left(a,c\right),sm3,abc}+{P^{\prime }}_{\left(b,c\right),sm3,abc}\right)}{3}$$

## Results and discussion

### Experimental setup

In our main experiments, we compare our two-stage approach (weighting with SSS, small complex extraction with Extract) against using the original PPI reliability (PPIREL) weighted network with standard clustering approaches to derive small complexes:

**Markov Cluster Algorithm (MCL) **[1] simulates stochastic flow to enhance the contrast between regions of strong and weak flow in the graph. The process converges to a partition with a set of high-flow regions (the clusters) separated by boundaries with no flow.

**Restricted Neighborhood Search Clustering (RNSC) **[2] is a local search algorithm that explores the solution space to minimize a cost function, calculated according to the number of intra-cluster and inter-cluster edges. RNSC first composes an initial random clustering, and then iteratively moves nodes between clusters to reduce the clustering's cost. It also makes diversification moves to avoid local minima. RNSC performs several runs, and reports the clustering from the best run.

**IPCA **[3] expands clusters from seeded vertices, based on rules that encode prior knowledge of the topological structure of protein complexes' PPI subgraphs. Whether a cluster is expanded to include a vertex is determined by the diameter of the resultant cluster and the connectivity between the vertex and the cluster.

**Clustering by Maximal Cliques (CMC) **[4] first generates all the maximal cliques from a given network, and then removes or merges highly overlapping clusters based on their inter-connectivity as follows. If the overlap between two maximal cliques exceeds a threshold *overlap thres*, then CMC checks whether the interconnectivity between the two cliques exceeds a second threshold *merge thres*. If it does, then the two cliques are merged; otherwise, the clique with lower density is removed.

**Clustering with Overlapping Neighborhood Expansion (ClusterONE) **[5] greedily expands clusters from seeded vertices to maximize a cohesiveness function, which is based on the edge weights within a cluster and the edge weights connecting the cluster to the rest of the network. It then merges highly-overlapping clusters.

**Proteins' Partition Sampler v2.3 (PPSampler2) **[6] partitions the PPI network into clusters using a Markov-chain Monte-Carlo approach to optimize an objective function. Novelly, it incorporates the size distribution of clusters in the objective function, and thus accounts for the sizes of complexes found in actual biological systems, where most of the complexes are small.

Any predicted complex with size greater than three is discarded. We run these algorithms with a range of values for their respective parameters, and select the settings that give the optimal performance for predicting small complexes. The parameter settings used in our experiments are given in Table 1.

**Table 1:** The six clustering algorithms and their parameters used for small complex discovery.

Clustering algorithm	Parameters
CMC	overlap thres = 1, merge_thres = 1
ClusterONE	*all default*
IPCA	-P1 -T0.4
MCL	-I 2
RNSC	-e10 -D50 -d10 -t20 -T3
PPSampler2	-f1DenominatorExponent 1 -f2

We also investigate the performance of using our SSS-weighted network with standard clustering approaches, and using the PPIREL network with our Extract approach.

We perform random sub-sampling cross-validation, repeated over ten rounds, using manually curated complexes as reference complexes for training and testing. For yeast, we use the CYC2008 [17] set which consists of 408 complexes, of which 259 are small (composed of two or three proteins). For human, we use the CORUM [18] set (filtered to remove duplicates and small complexes that are subsets of large ones), which consists of 1352 complexes, of which 701 are small. In each cross-validation round, *t*% of the complexes (large and small) are selected for testing, while all the remaining complexes are used for training. Each edge (*a, b*) in the network is given a class label *lg-comp *if *a *and *b *are in the same large training complex; otherwise it is labeled *sm-comp *if *a *and *b *are in the same small training complex; otherwise its class label is *non-comp*. Learning in SSS is performed using these labels, and the edges of the network are weighted using the learned models. Small complexes are then extracted from the weighted network. The predicted complexes are evaluated by matching them with only the small test complexes.

We design our experiments to simulate a real-use scenario of complex prediction in an organism where a few complexes might already be known, and novel complexes are to be predicted: in each round of cross-validation, the training complexes are those that are known and leveraged for learning to discover new complexes, while the test complexes are used to evaluate the performance of each approach at this task. Thus we use a large percentage of test complexes *t *= 90%. In yeast, this gives about 233 small test complexes and 26 small training complexes per cross-validation iteration; in human, this gives about 631 small test complexes and 70 small training complexes.

### Evaluation methods

We use precision-recall graphs to evaluate the predicted clusters, by matching the generated clusters with the reference test complexes, and calculating recall (sensitivity) and precision. We require a generated cluster to be identical to a complex to be considered a correct match. Each cluster *P *is ranked by its score, which is either the cohesiveness-weighted density (for Extract), or weighted density (for other clustering algorithms). To obtain a precision-recall graph, we calculate and plot the precision and recall of the predicted clusters at various cluster-score thresholds. Given a set of predicted clusters *P *= {*P*1*, P*2*, . . *.}, a set of test reference complexes *C *= {*C*1*, C*2*, . . *.}, and a set of training reference complexes *T *= {*T*1*, T*2*, . . *.}, the recall and precision at score threshold *d *are defined as follows:

$$Recall_{d}=\frac{\left|\left\{C_{i}|C_{i}\in C\wedge \exists P_{j}\in P,score\left(P_{j}\right)\geq d,P_{j}matches\;C_{i}\right\}\right|}{\left|C\right|}$$

$$Precision_{d}=\frac{\left|\left\{P_{j}|P_{j}\in P,score\left(P_{j}\right)\geq d\wedge \exists C_{i}\in C,C_{i}matches\;P_{j}\right\}\right|}{\left|\left\{P_{k}|P_{k}\in P,score\left(P_{k}\right)\geq d\wedge ∄T_{i}\in T,T_{i}matches\;P_{k}\right\}\right|}$$

The precision of clusters is calculated only among those clusters that do not match a training complex, to eliminate the bias of the supervised approach (SSS) for predicting training complexes well. As a summarizing statistic of a precision-recall graph, we also calculate the area under the curve (AUC) of a precision-recall graph.

To measure the quality of a predicted complex, we derive the semantic coherence of its Gene Ontology (GO [19]) annotations across the three GO classes, biological process (BP), cellular compartment (CC), and molecular function (MF). First, we derive the BP semantic similarity between two proteins as the information content of their BP annotations' most informative common ancestor [20]. Then we define the BP semantic coherence of a predicted complex as the average BP semantic similarity between every pair of proteins in that complex (likewise for CC and MF).

### Prediction of small complexes

**Figure 2:**
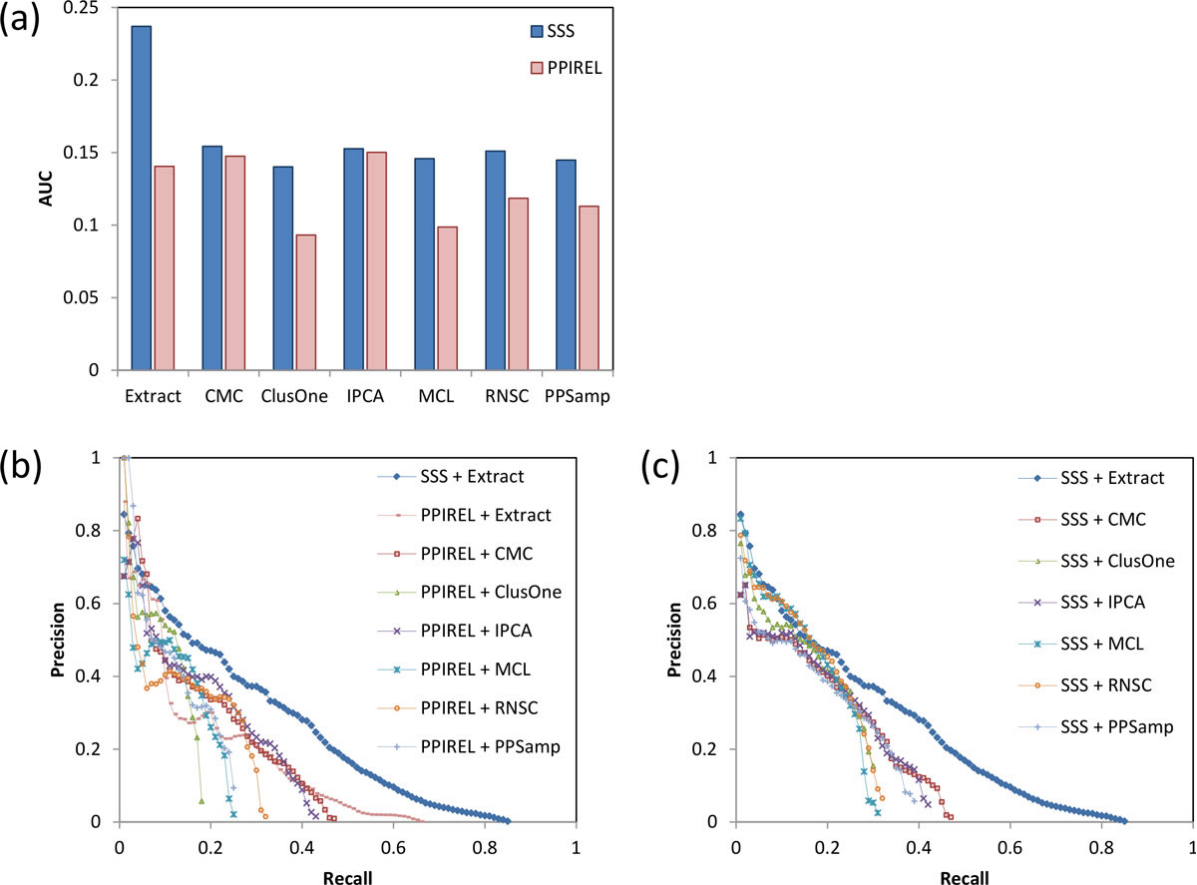
**Performance of small complex prediction in yeast, (a) precision-recall AUC, (b) and (c) precision-recall graphs**.

In this section we compare the performance of small complex prediction using our weighting approach (SSS) versus PPI reliability (PPIREL), and using our complex extraction algorithm (Extract) versus other clustering algorithms (CMC, ClusterOne, IPCA, MCL, RNSC, PPSampler2). Figure 2a shows the performance of prediction of yeast small complexes, in terms of precision-recall AUC. Our 2-stage approach (SSS + Extract) outperforms all other approaches tested here, including using the PPIREL or SSS-weighted networks with standard clustering algorithms, or the PPIREL-weighted network with Extract. Furthermore, when using standard clustering algorithms to discover small complexes, weighting the network with SSS gives improved performance compared to using PPIREL (especially for ClusterOne, MCL, RNSC, and PPSampler2).

Figure 2b shows the precision-recall graphs comparing our approach (SSS + Extract) to the baselines of standard clustering algorithms applied on a PPIREL network. While our approach has lower precision among the initial top predictions (at recall less than 5%), beyond that we attain substantially greater precision: for example, at 40% recall, our approach attains more than three times the precision than the other clustering approaches (28% versus 9%). Furthermore, we attain substantially higher recall as well. Figure 2c shows the precision-recall graphs when the standard clustering algorithms are applied on the SSS-weighted network. Using the SSS-weighted network, most of the clustering algorithms achieve improved precision in the mid-recall ranges, as well as gains in recall. However, our approach (SSS + Extract) still maintains greater precision in most of the recall range.

**Figure 3:**
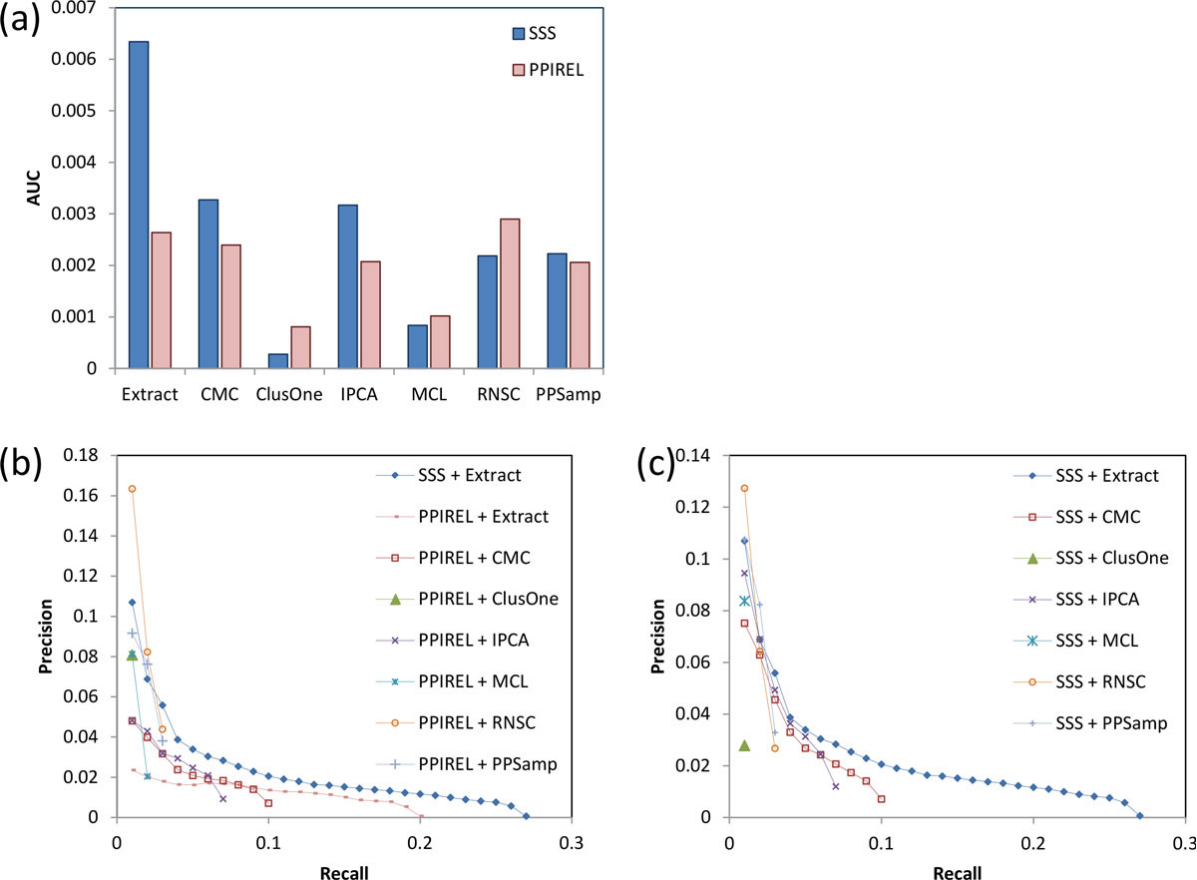
**Performance of small complex prediction in human, (a) precision-recall AUC, (b) and (c) precision-recall graphs**.

Figure 3 shows the performance of prediction of human small complexes. The prediction of complexes in human is much more challenging than in yeast, so the AUCs achieved here are correspondingly lower. Nonetheless, our approach (SSS + Extract) still outperforms all the other approaches, including using the PPIREL or SSS-weighted networks with standard clustering algorithms, or the PPIREL-weighted network with Extract. When using standard clustering algorithms to discover small complexes, weighting the network with SSS gives improved performance only for CMC and IPCA, while performance remains the same or decreases for the other clustering algorithms.

Figure 3b and 3c show the corresponding precision-recall graphs. As in yeast, our approach (SSS + Extract) outperforms the standard clustering algorithms applied on the PPIREL-weighted network by achieving substantially higher recall, as well as greater precision in almost the whole recall range (Figure 3b). Using the SSS instead of the PPIREL-weighted network, CMC and IPCA achieve higher precision, while the other clustering algorithms suffer from lower precision or recall (Figure 3c).

In the following section we investigate how the various techniques incorporated in SSS and Extract improve the performance of small complex prediction.

### How do SSS and Extract improve performance?

**Figure 4:**
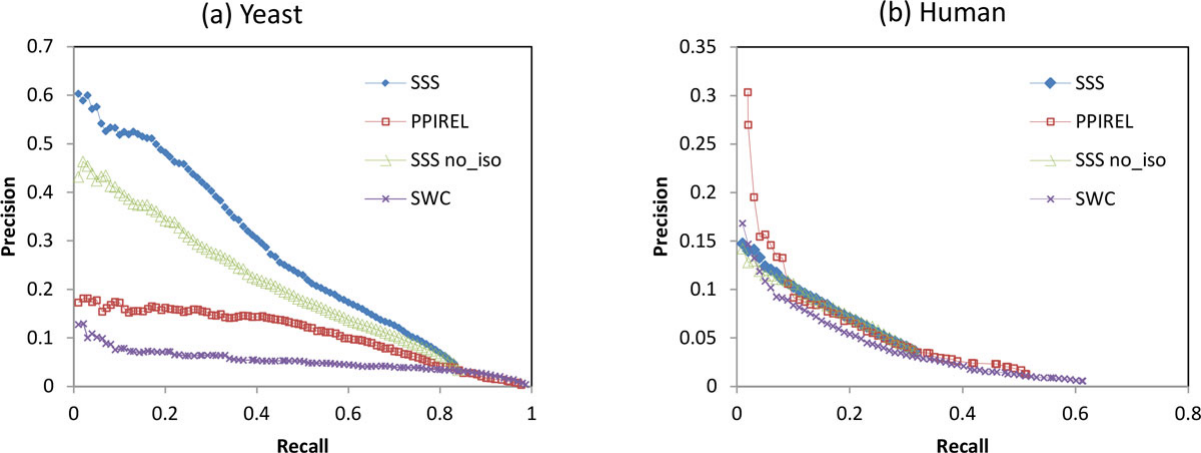
**Performance of classification of small complex edges, in (a) yeast, (b) human**.

Figures 2 and 3 showed that weighting the network with SSS improves yeast small complex prediction in four of six clustering algorithms, while it only improves human complex prediction in two clustering algorithms. To investigate the benefits of SSS weighting, we compare the performance of the weighting approaches in *classifying *edges as belonging to small complexes. Each weighting approach is used to weight the edges of the network, and the precision-recall graph is obtained by varying a threshold on the edge weights. Figure 4a shows the precision-recall graph for classification of yeast small complex edges. SSS achieves much higher precision than classifying by PPIREL, as the SSS weights more accurately reflect membership in small complexes. This leads to improved performance by clustering algorithms when applied to the SSS-weighted network to predict small yeast complexes. On the other hand, when classifying edges in small human complexes, Figure 4b shows that SSS has lower precision than PPIREL at the lower recall range, with only similar or marginally better precision at higher recall ranges. Thus, only two clustering algorithms obtain improved performance from clustering the SSS-weighted network. Figure 4 also shows the poor performance of a previously proposed supervised weighting approach SWC [8], which learns a model for all co-complex edges in general, as opposed to distinct models for small and large complexes. As the number of edges in a complex grows quadratically with its number of proteins, the edges from large complexes far outnumber those from small complexes, so SWC's learned model reflects the characteristics of large complexes. Thus, SWC suffers from poor performance in classifying edges from small complexes, demonstrating the importance of the size-specific modeling of SSS.

**Figure 5:**
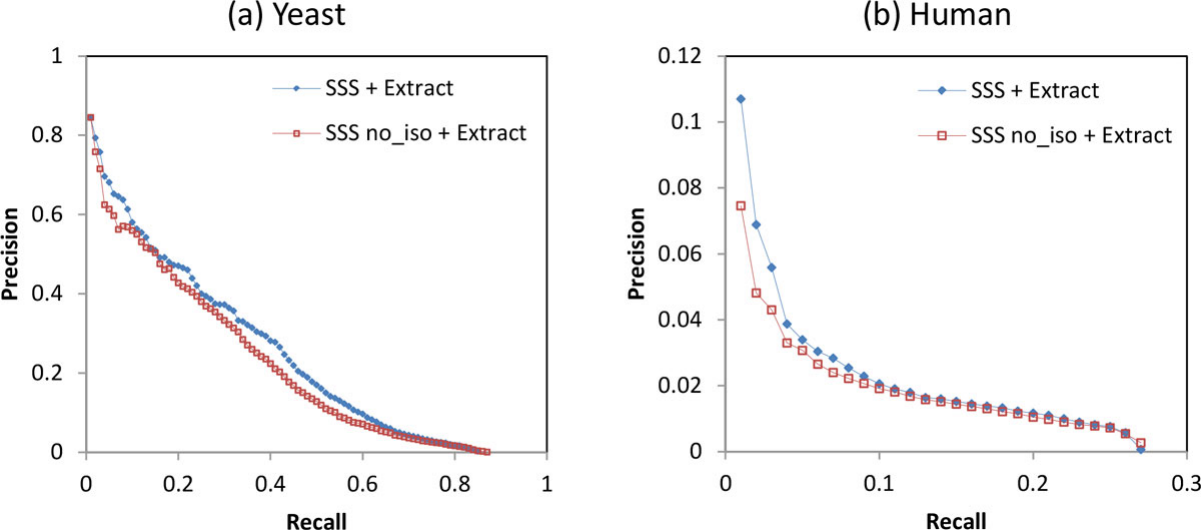
**Performance of small complex prediction with and without isolatedness feature in SSS, in (a) yeast, (b) human**.

The *SSSno_iso *graph in Figure 4 shows that if the isolatedness feature is not incorporated into SSS (in other words, steps 4 to 6 of SSS are skipped), precision drops substantially in yeast, showing the utility of the isolatedness function in predicting small complex edges. However, in human, incorporating the isolatedness feature gives only marginal improvement in precision. Figure 5 shows the performance of small complex prediction, when SSS is used with and without the isolatedness feature, with the complexes derived by Extract. Incorporating isolatedness gives a noticeable boost to precision in both yeast and human, demonstrating that isolatedness benefits the prediction of small complexes by improving the SSS weighting of edges.

**Figure 6:**
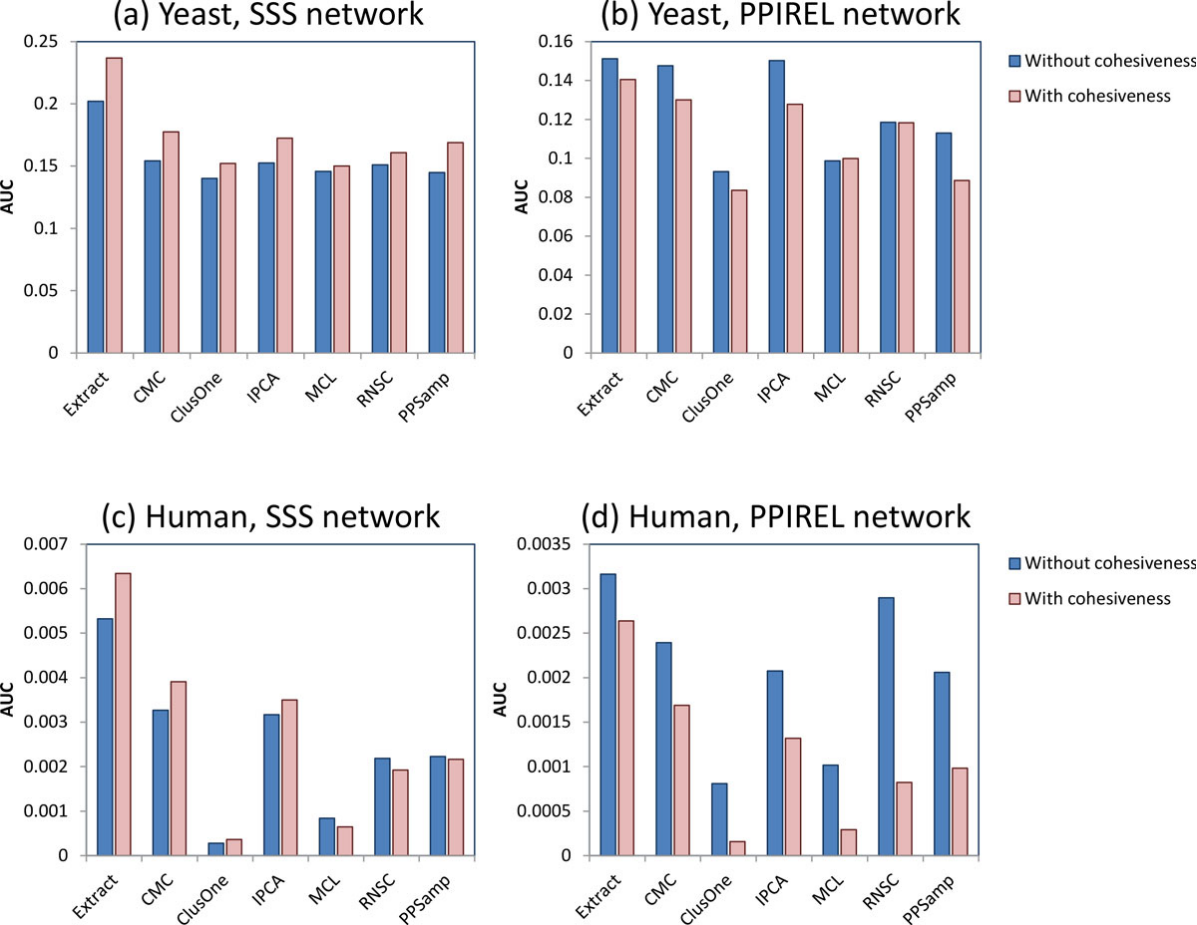
**Performance of small complex prediction with and without cohesiveness weighting for scoring clusters, for (a) SSS network in yeast, (b) PPIREL network in yeast, (c) SSS network in human, (d) PPIREL network in human**.

Figures 2 and 3 showed that using Extract to derive small complexes from the PPIREL network does not perform better than using most of the other clustering algorithms (Extract achieves higher recall, at the expense of precision). We investigate the effect of cohesiveness weighting in Extract, applied on the SSS network versus the PPIREL network. Figure 6a shows the performance of the clustering algorithms applied on the SSS network, with and without scoring by cohesiveness weighting, for predicting yeast small complexes. For Extract (where cohesiveness weighting is used by default), scoring without cohesiveness weighting means a cluster's score is its weighted density. For the other clustering algorithms (where weighted density is used by default), scoring with cohesiveness weighting means a cluster's score is the product of its weighted density and its cohesiveness (ratio of sum of internal edges over internal and outgoing edges). With the SSS network, scoring by cohesiveness weighting improves performance across all clustering algorithms. On the other hand, Figure 6b shows that, with the PPIREL network, scoring by cohesiveness weighting decreases performance across most clustering algorithms. Thus, cohesiveness weighting appears useful only when edges are weighted using SSS.

Figure 6c and 6d show the corresponding charts for human complexes, with and without cohesiveness weighting. With the SSS network, cohesiveness weighting improves performance in four of seven clustering algorithms; whereas with the PPIREL network, cohesiveness weighting decreases performance in all clustering algorithms. Thus, in human complexes as well, cohesiveness weighting appears useful only when edges are weighted using SSS.

### Example complexes

In this section we present some example complexes that are difficult to predict using the PPIREL network with any standard clustering algorithm, but can be predicted with our approach (SSS + Extract). Since the various clustering approaches output different numbers of predictions, we consider only the top scoring predicted clusters with a cross-validation precision level greater than some threshold. For yeast we use a precision threshold of 10%, but for human we use a lower precision threshold of 2%, since fewer human complexes are predicted with high precision.

**Figure 7:**
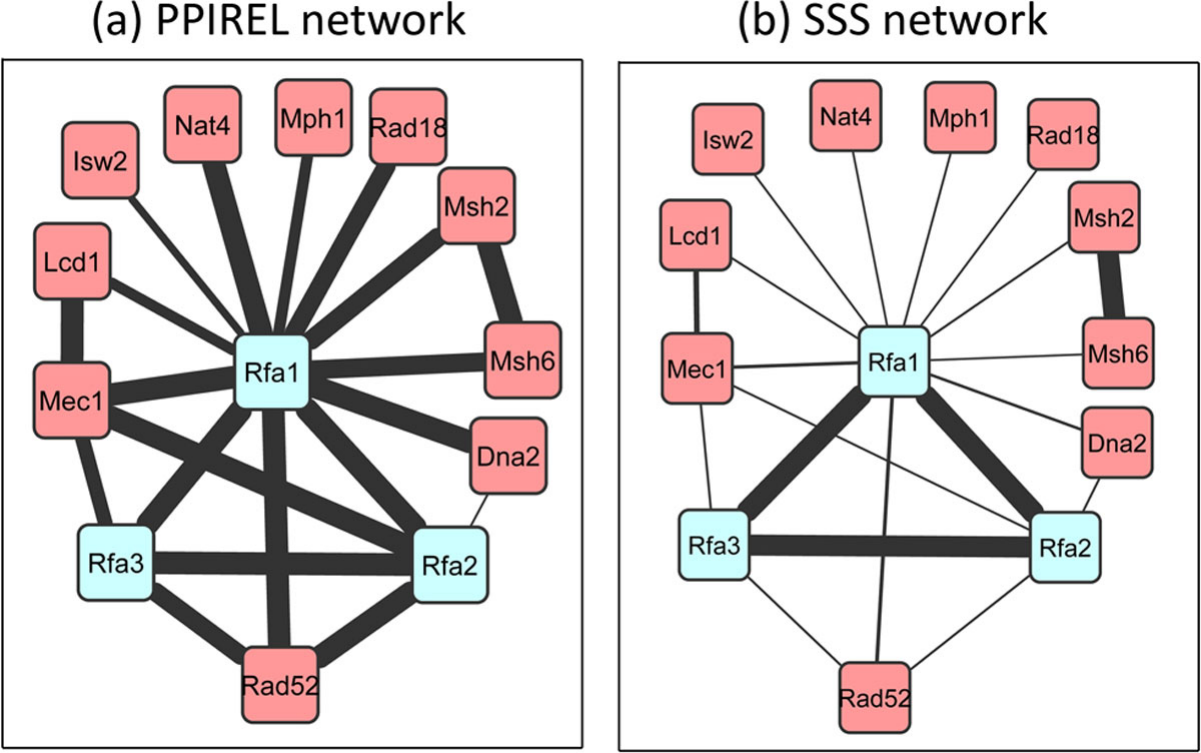
**DNA replication factor A complex in yeast, in (a) PPIREL network, (b) SSS network**.

The DNA replication factor A complex in yeast consists of three proteins, Rfa1p, Rfa2p, and Rfa3p. Figure 7a shows the PPIREL network around this complex, with edge widths scaled to PPI reliability scores. The complex is embedded within two size-4 cliques (with Rad52p, and Mec1p), with high PPIREL weights. Moreover, Rfa1p is also connected via high PPIREL weights to many external proteins, some of which form size-3 cliques as well. As a result, none of the standard clustering algorithms applied on the PPIREL network predicted this complex, in any cross-validation round. Figure 7b shows the SSS network, with edge widths scaled to the small co-complex posterior probability scores. The three proteins in the complex remain interconnected with high edge weights, while the extraneous edges' weights are now markedly lowered. Thus, our Extract algorithm is able to retrieve this complex from the SSS network consistently across all cross-validation rounds where it is tested.

**Figure 8:**
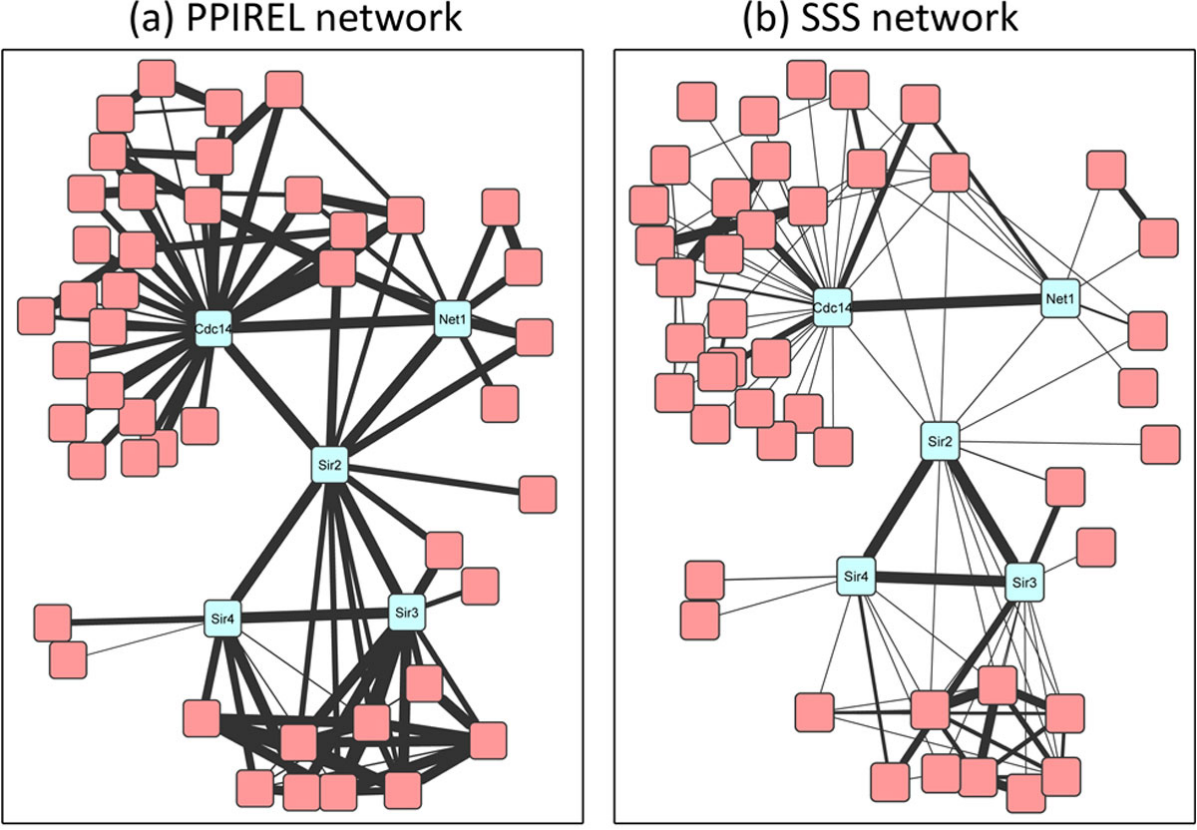
**Chromatin silencing complex and RENT complex in yeast, in (a) PPIREL network, (b) SSS network**.

Figure 8 shows two yeast complexes, with an overlapping protein (Sir2p), involved in transcriptional silencing: the chromatin silencing complex, consisting of Sir2p, Sir3p, and Sir4p, and the RENT complex, consisting of Sir2p, Cdc14p, and Net1p. In the PPIREL network (Figure 8a), each of the two complexes are connected via highly-weighted extraneous edges to many external proteins. Once again, none of the standard clustering algorithms applied on the PPIREL network could predict either of these complexes, in any cross-validation round. In the SSS network (Figure 8b), the chromatin silencing complex remains connected with high edge weights, with a marked reduction in the weights of the extraneous edges. Thus our Extract algorithm retrieves this complex from the SSS network consistently across all cross-validation rounds where it is tested. On the other hand, in the RENT complex, the weights of two edges (from Sir2p to the other two proteins) are now even lower than some of its extraneous edges. As a result, our Extract algorithm retrieves this complex in only 33% of the cross-validation rounds where it is tested. Nonetheless, this is still an improvement over using the PPIREL network with standard clustering algorithms.

**Figure 9:**
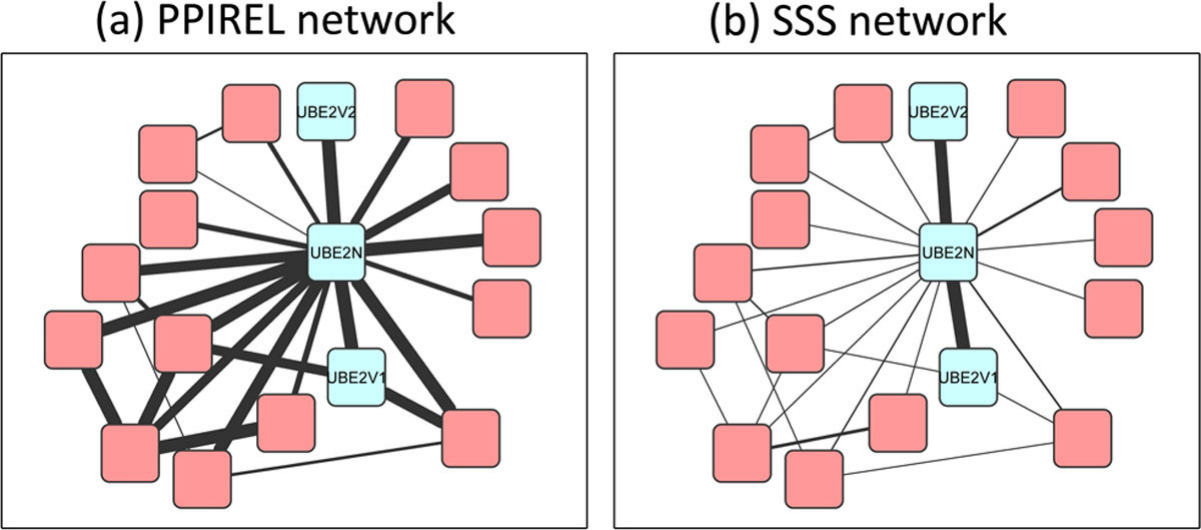
**Two human ubiquitin ligase complexes, in (a) PPIREL network, (b) SSS network**.

Figure 9 shows two human ubiquitin ligase heterodimer complexes with an over-lapping protein: the UBE2V1-UBE2N and UBE2V2-UBE2N complexes. In the PPIREL network (Figure 9a), UBE2N is connected via highly-weighted edges to many other external proteins, forming a number of size-3 cliques with them. The UBE2V1-UBE2N complex is embedded within two size-3 cliques, making it difficult to discover: none of the standard clustering algorithms predicted this complex in any cross-validation round. On the other hand, the UBE2V2-UBE2N complex is relatively isolated as UBE2V2 is not connected to any other external protein, allowing CMC and IPCA to predict this complex consistently. In our SSS network (Figure 9b), all extraneous edges' weights have been dramatically lowered, leaving the co-complex edges with high weights. Thus our Extract algorithm retrieved UBE2V1-UBE2N 78% of the time, and UBE2V2-UBE2N 100% of the time.

### Quality of novel complexes

In this section we compare the number and quality of high-confidence novel complexes predicted by our approach (SSS + Extract), against using standard clustering algorithms on the PPI reliability network. When weighting the network with SSS, the entire set of reference complexes is used for training. We filter the predicted complexes to remove those that match any reference complex, and to keep only high-confidence predictions: the score of each predicted complex is mapped to a precision value, using the cross-validation results, and only predicted complexes with estimated precision greater than a confidence threshold are kept. For yeast, this confidence threshold is 0.5; for human, a lower threshold of 0.1 is used, since much fewer complexes are predicted with high precision.

**Figure 10:**
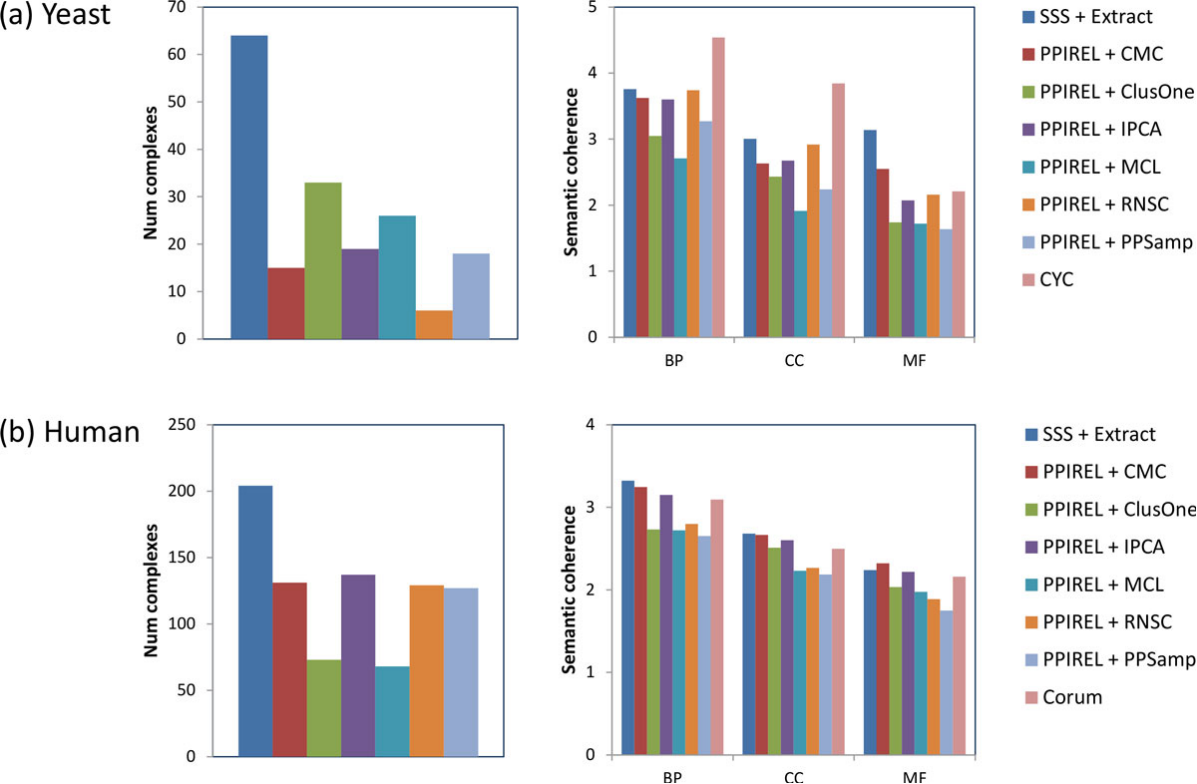
**Number of high-confidence novel predictions, and their semantic coherences, in (a) yeast, (b) human**.

Figure 10a shows the number of high-confidence novel complexes predicted in yeast, and their average BP, CC, and MF semantic coherence, using the different approaches. Compared to the other approaches, SSS with Extract generates more than twice as many high-confidence novel predictions, with equal or greater quality: our predicted complexes have greater coherence than ClusterOne, MCL, or PPSampler2 (*p <*.05 in at least one of BP, CC, or MF), and similar coherence with the other approaches. The CYC2008 reference complexes have much higher BP and CC coherence, but lower MF coherence.

Figure 10b shows the corresponding charts for human predictions. Again, our approach generates more high-confidence novel predictions than the other approaches, with equal or greater quality: our predicted complexes have greater coherence than ClusterOne, MCL, RNSC, or PPSampler2 (*p <*.05 in at least one of BP, CC, or MF), and similar coherence with the other approaches. Our predicted complexes have similar semantic coherence compared to the Corum reference complexes.

Finally, we briefly mention two novel complexes, predicted by our approach, that we have validated via a literature scan. Our approach predicts a high-scoring yeast cluster consisting of Cap1p and Cap2p, which is not found in our reference database of complexes. However, a literature scan revealed this to be the capping protein heterodimer, which binds to actin filaments to control filament growth [21]. Our approach also predicts a novel high-scoring human cluster consisting of PKD1 and PKD2. A literature scan revealed that these two proteins, which are involved in autosomal polycystic kidney disease, have been found to form a PKD1-PKD2 heterodimer [22].

## Conclusion

The size of protein complexes has been noted to follow a power distribution, meaning that a large majority of complexes are small (consisting of two or three distinct proteins). Thus the discovery of small complexes is an important subtask in protein complex prediction. Predicting small complexes from PPI networks is inherently challenging. Small groups of proteins are likelier to take on topological characteristics of real complexes by chance: for example, fully dense groups of two or three proteins correspond to edges or triangles respectively, but only a few of these actually correspond to small complexes. Furthermore, the prediction of small complexes is especially susceptible to noise (missing or spurious interactions) in the PPI network, as these can easily disconnect a small complex, or embed it within a larger clique.

We propose a two-stage approach, SSS and Extract, for discovering small complexes. First, the PPI network is weighted by size-specific supervised weighting (SSS), which integrates heterogeneous data and their topological features with an overall topological isolatedness feature, and uses a naive-Bayes maximum-likelihood model to weight the edges with their posterior probabilities of being in a small complex, and in a large complex. Integrating other data sources into the PPI network can help reduce noise, while incorporating the topological features across multiple data sources makes it less likely that random protein groups take on topological characteristics of complexes by chance. In our second stage, Extract, the SSS-weighted network is analyzed to extract putative small complexes and score them by cohesiveness-weighted density, which incorporates both small-co-complex and large-co-complex weights of internal and outgoing edges. This reduces the impact of noisy edge weights in deriving reliable scores for predictions, as more edge weights around the candidate complex are utilized.

While a few previous approaches have used supervised learning to weight PPI edges, none of them have done so in a complex-size-specific manner, or incorporated isolatedness as a feature in this way. Our adaptation of cohesiveness to address the problem of the small number of edge weights available in scoring small complexes is also novel.

We test our approach on the prediction of yeast and human small complexes, and demonstrate that our approach outperforms some commonly-used clustering algorithms applied on a PPI reliability network, attaining higher precision and recall. Furthermore, our approach generates a greater number of novel predictions with higher quality in terms of Gene Ontology semantic coherence. Nonetheless, the performance of small complex prediction still lags behind that of predicting large complexes, especially for human complexes.

We note that a significant challenge for human complex prediction is insufficient PPI data. An estimate of the human interactome size is around 220, 000 PPIs [23]. Our human PPI data consists of around 140, 000 PPIs, and with an estimated false-positive rate of 50%, this means that our human PPI network represents only a third of the true human PPI network. In comparison, in yeast an estimate of the interactome size is around 50, 000 PPIs. Our yeast PPI data consists of around 120, 000 PPIs, so even with an estimated false-positive rate of 50%, our yeast PPI network can be believed to be a good representation of the actual yeast PPI network. The much poorer representation of the true human interactome partially explains the poorer performance of our approach on human complexes.

Nonetheless, there is still room for further work on complex detection techniques that may improve the prediction of small human complexes. A possible future direction is to adapt other techniques that have proved useful for large complex prediction, such as GO term decomposition and hub removal [24], which might further improve the performance of small complex prediction.

## Competing interests

The authors declare that they have no competing interests.

## Authors' contributions

CHY derived and implemented the algorithms and drafted the manuscript. CHY and OM designed and performed the experiments. LW conceived and directed the study. All authors read and approved the final manuscript.

## Declarations

This work is supported in part by a Singapore Ministry of Education grant MOE2012-T2-1-061 and a National University of Singapore NGS scholarship. Publication charges for this article is funded in part by a Singapore Ministry of Education grant MOE2012-T2-1-061.

This article has been published as part of *BMC systems Biology *Volume 8 Supplement 5, 2014: Proceedings of the 25th International Conference on Genome Informatics (GIW/ISCB-Asia): Systems Biology. The full contents of the supplement are available online at http://www.biomedcentral.com/bmcsystbiol/supplements/8/S5.
